# Language aptitude is related to the anatomy of the transverse temporal gyri

**DOI:** 10.1007/s00429-024-02883-4

**Published:** 2024-12-19

**Authors:** Carmen Ramoser, Aileen Fischer, Johanneke Caspers, Niels O. Schiller, Narly Golestani, Olga Kepinska

**Affiliations:** 1 Brain and Language Lab, Vienna Cognitive Science Hub, University of Vienna, Vienna, Austria; 2 Max Planck School of Cognition, Max Planck Institute for Human Cognitive and Brain Sciences, Leipzig, Germany; 3 Faculty of Humanities, Leiden University Centre for Linguistics, Leiden University, Leiden, Netherlands; 4 Department of Linguistics and Translation, City University of Hong Kong, Kowloon Tong, Hong Kong SAR; 5 Brain and Language Lab, Department of Psychology, Faculty of Psychology and Educational Sciences, University of Geneva, Geneva, Switzerland; 6 Department of Behavioral and Cognitive Biology, Faculty of Life Sciences, University of Vienna, Vienna, Austria

**Keywords:** Auditory cortex morphology, Heschl’s gyrus, Transverse temporal gyrus, Language aptitude, Foreign language learning

## Abstract

Why is it that some people seem to learn new languages faster and more easily than others? The present study investigates the neuroanatomical basis of language learning aptitude, with a focus on the multiplication pattern of the transverse temporal gyrus/gyri (TTG/TTGs) of the auditory cortex. The size and multiplication pattern of the first TTG (i.e., Heschl’s gyrus; HG) and of additional posterior TTGs, when present, are highly variable both between brain hemispheres and individuals. Previous work has shown the multiplication pattern of the TTGs to be related to musical and linguistic abilities. Specifically, one study found that high language learning aptitude correlated with more TTGs in the right hemisphere, even though language functions are generally left-lateralized. In this study, we used the recently developed TASH (Toolbox for the Automated Segmentation of Heschl’s Gyrus) and MCAI (Multivariate Concavity Amplitude Index) toolboxes to automatically extract structural (e.g., cortical volume, surface area, thickness) and multiplication pattern measures of the TTGs from 82 MRI scans, and related them to participants’ language aptitude scores. In contrast to previous results, we found that higher language aptitude was related to *fewer* TTGs in the right hemisphere and to greater surface area of the first right TTG and of the second left TTG. Furthermore, more languages learned in life were associated with higher language learning aptitude, opening up questions about the structure–function relationship of the TTGs and language learning, and about how language aptitude and language learning are related.

**Supplementary Information:**

The online version contains supplementary material available at 10.1007/s00429-024-02883-4.

## Introduction

Individuals differ in their rate and success in learning a foreign language (L2). Apart from age and motivation, these are largely determined by foreign language aptitude, or simply language aptitude (Carroll 1981; Dörnyei and Skehan 2003; Turker et al. 2017; Wen et al. 2017). The ‘founding father’ of language aptitude research, John Carroll, viewed aptitude as the capacity to learn languages fast and with facility, with this ability being relatively stable and innate (Carroll 1981). He conceptualised language aptitude as consisting of four distinct and measurable abilities: phonemic coding ability (capacity to code unfamiliar sounds so that they can be retained), grammatical sensitivity (capacity to identify the functions that words fulfil in sentences), inductive language ability (capacity to extrapolate from a given corpus to create new sentences) and associative memory (capacity to form associative links in memory) (Carroll 1981; Dörnyei and Skehan 2003). These categories have mostly held up in empirical investigations since then, but due to their similarity, ‘grammatical sensitivity’ and ‘inductive language learning’ have been combined under the concept of ‘language analytic ability’ (Biedroń, 2015; Biedroń and Pawlak 2016; Skehan 2002; Turker et al. 2017, 2019; Wen et al. 2017). Current views consider language aptitude to be more dynamic than it was viewed in Carroll’s time; for example, it is recognised that it changes with age (Robinson 2007; Wen et al. 2017). Some studies show that elective multilinguals have higher aptitude scores (Eisenstein 1980; Grigorenko et al. 2000; Sparks et al. 1995), which could be due to increased meta-linguistic awareness, while others do not (Harley and Hart 1997; Sawyer 1992). Higher language aptitude in elective multilinguals could however also be due to a stable aptitude creating intrinsic motivation to seek out opportunities to learn languages. In fact, twin studies on instructed second language learning in school children and young adults estimated a heritability of 67–72% (67% in Dale et al. 2010; 71% in Vinkhuyzen et al. 2009; 72% in Coventry et al. 2012). In this case, active gene environment interaction (when one selects certain environments following their genetic predispositions, also referred to as niche-picking) (Hart et al. 2021), can lead to individuals with high language aptitude to seek out language courses, language-related disciplines at university, or interpreter’s/translator’s careers. For a neurofunctional model of how intrinsic motivation can improve word learning by synaptic dopamine signaling see Ripollés et al. (2018).

**Fig. 1:**
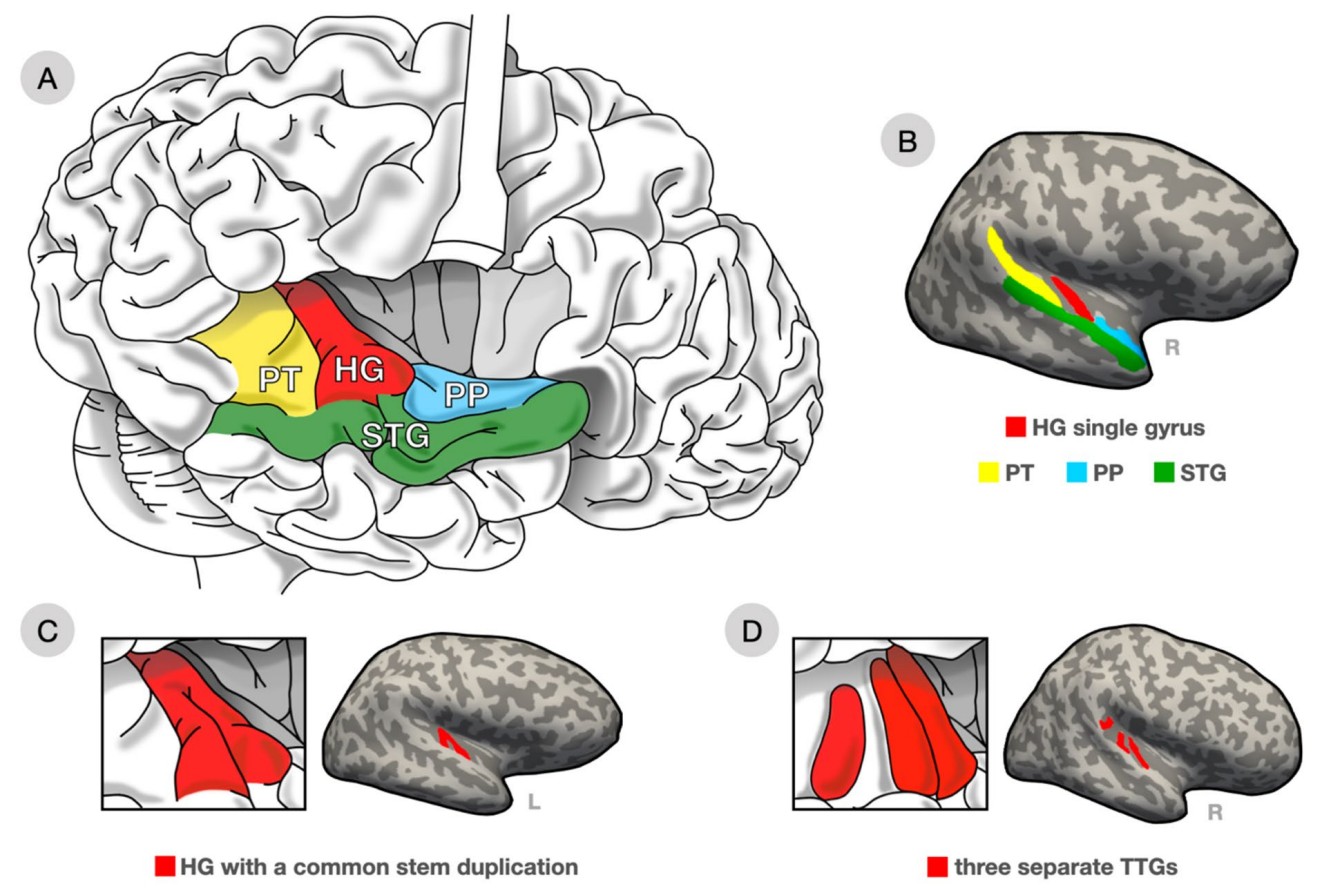
The anatomy of the auditory cortex and examples of different types of TTGs from the present data set. **A** Illustration of the location of the HG (1st TTG) within the broader regions of the auditory cortex (*PP* planum polare, *PT* planum temporale, *STG* superior temporal gyrus, lateral aspect). **B** The same regions overlaid on inflated surface of freesurfer-processed T1 MRI image of one of the participants with a single HG. **C** Illustration and example of a common stem duplication. **D** Illustration and example of three separate TTGs. Artwork by Patricia Jaqueline Matic

The question arises whether individual differences in language aptitude are reflected in differences in brain structure. The present study focuses on the connection between language aptitude and the structure of the auditory cortex, given its known role in processing auditory input such as speech, music, and environmental sounds (Moerel et al. 2014), and specifically on the transverse temporal gyrus/gyri (TTG/TTGs) in the Sylvian fissure. The TTG(s) are located on the superior plane of the superior temporal gyrus (STG) within the Sylvian fissure. Early auditory cortex, or the primary auditory cortex, tends to lie within the medial two-thirds of the first TTG, also known as Heschl’s gyrus (HG) (Rademacher et al. 1993). Posterior to HG lies the planum temporale (PT), which includes secondary auditory cortex. Both HG and PT show leftward structural asymmetry in healthy populations, consistent with the left-hemisphere specialisation for language (Geschwind and Levitsky 1968; Marie et al. 2015; Moerel et al. 2014; Penhune et al. 1996). HG and PT are separated by the first Heschl’s sulcus (HS), and additional TTGs, if present, belong to the PT (Marie et al. 2015; Penhune et al. 1996; Rademacher et al. 1993). We use the term multiplication pattern for the number (duplication, triplication, …) and shape of the TTG. TTG morphology (i.e. surface area, thickness, and volume) and multiplication patterns are highly variable both between individuals and between hemispheres (Marie et al. 2015; Rademacher et al. 1993). A sulcus intermedius (SI) may be present, dividing HG incompletely. If the SI divides the HG at the lateral but not the medial end, this is called a common stem duplication (CSD). According to older definitions, the posterior part belongs to the PT if the SI is more than half the length of HG (Golestani et al. 2007; Penhune et al. 1996). Other studies have always assigned the posterior branch of the CSD to HG (Schneider et al. 2005; Seither-Preisler et al. 2014; Wengenroth et al. 2014). Additional TTGs are typically referred to as duplicated (or triplicated) HGs, although by definition they are not HG (see Fig. 1 for examples). In general, the multiplication pattern of the TTG is thought to develop in utero (Chi et al. 1977; López Ramón y Cajal 2019), be stable during development (Seither-Preisler et al. 2014), and be heritable, as shown in studies of di- and monozygotic twins (Peper et al. 2007).

The neural processing of language is left-hemisphere dominant in most individuals. A growing body of work shows that there are relationships between the anatomy of HG and/or of the TTGs and language. For example, more successful learners of foreign speech sounds and linguistic pitch patterns have larger left HG volumes and more left TTGs (Golestani et al. 2007; Wong et al. 2008). The same was found for phonetic transcription expertise (Golestani et al. 2011). Studies have also linked dyslexia to additional TTGs in the right hemisphere in children (Altarelli et al. 2014; Serrallach et al. 2016) and the left hemisphere in adults (Leonard et al. 2001). A recent large study on dyslexia and the auditory cortex found a significant positive correlation between left first TTG surface area and left TTGs multiplication patterns and better word reading in children (Blockmans et al. 2023).

Recent findings regarding language aptitude contrast with the left-hemispheric dominance of language. Individuals with higher language aptitude and higher phonetic coding abilities were more likely to have additional TTGs in the *right* hemisphere (Turker et al. 2017, 2019). Turker et al. (2019) found an association between higher language aptitude scores and CSDs in the right hemisphere. In another study, participants with high language analytic abilities, based on their performance in an artificial grammar learning task, showed more widespread activation generally, and higher right-hemispheric activity specifically, compared to those with average scores (Kepinska et al. 2017a). An EEG study further showed that highly skilled learners exhibited stronger local synchronisation within the right hemisphere during an artificial grammar learning task (Kepinska et al. 2017c). A recent review linking individual right-hemisphere differences to language learning proposed a model in which the right hemisphere integrates information across modalities and extracts relevant features that the left hemisphere learns to process reliably (Prat et al. 2023). As a result, early language learning would be facilitated by the right hemisphere, whereas later right-hemisphere activation "might reflect poorer performance, more reliance on context, or a slower transition to feature-specific LH systems" (p. 4).

Turker et al. (2019) found that not only phonetic processing but overall language aptitude is related to TTG anatomy, implying higher level processing. This is supported by findings of causal perceptual decision-making in the auditory cortex that enhances task performance (Francis et al. 2018; Tsunada et al. 2016). More importantly, activity in the auditory cortex is related to auditory working memory (Huang et al. 2016; Kumar et al. 2016) and the close connection between language aptitude and working memory is well known in the literature: The phonological loop supports vocabulary acquisition in the first and second language and possibly long-term grammar acquisition (Baddeley 2003; Baddeley et al. 1998; Duyck et al. 2003) and indeed polyglots show expanded phonological loop capacity, while no other comparable cognitive task proved different to controls (Papagno and Vallar 1995). In a study on the structural connectivity underlying language aptitude it was found that grammatical inferencing, vocabulary learning and verbal working memory is related to connections from the temporal regions to inferior frontal regions (Xiang et al. 2012). In this study we wanted to further elucidate if a relationship between the high-level construct of language aptitude and the structure of the auditory cortex can be found.

To investigate the relationship between language aptitude and TTG anatomy, we extracted structural measures (e.g. volume, surface area, etc.) and multiplication patterns of HG and additional TTGs, if present, from healthy adults and related them to their language aptitude. Based on previous findings, our main prediction was that participants with higher language aptitude would have higher multiplications, even partial, of the first and/or of additional TTGs in the right hemisphere.

If language aptitude is a stable innate predisposition as originally proposed by Carroll (1981), it could be that it does not correlate with the number of languages learned. In case that it does, this could be either due to niche construction or experience-driven development of expertise in aptitude mediated by plasticity of the auditory cortex. The latter is supported by a study that associated bilingualism with a higher volume of bilateral HG (Ressel et al. 2012), and a more recent study in a group of individuals who varied widely in their multilingual language experience that showed a relationship between the thickness of the second TTG and multilingualism (Kepinska et al. 2023).

To investigate this, we additionally related the relatively diverse multilingual experience of our participants to language aptitude and to auditory cortex anatomy. While it is not easy to distinguish the underlying process in case there is a relationship between multilingualism and language aptitude, Grasby et al. (2020) found that genetic and prenatal influences affect more strongly cortical surface area, while regulatory elements in the adult brain influence more cortical thickness. If we can correlate increased multilingualism with TTG surface area or its similarly genetically determined multiplication pattern, this would support effects of niche construction. On the other hand, if a relationship to cortical thickness is found, this could indicate that language aptitude is malleable. Due to previous literature, we expect to find this.

## Methods

### Language aptitude

Language aptitude was assessed with the LLAMA test (Meara 2005). The LLAMA test is free, computer-based, and language-independent, relying only on pictures and on a made-up language based on Native American languages. It consists of four subtests:

1. LLAMA_B tests vocabulary learning, where participants have 2 min to learn 20 pairs of words and imaginary figures. Afterwards, they are asked to identify the correct figure for each word.
2. LLAMA_D tests phonetic memory. Participants hear a stream of non-words and then the same non-words interspersed with new non-words. For every item, they have to decide if they already heard it or not.
3. LLAMA_E tests sound-symbol correspondence. Participants have 2 min to learn associations between a consonant–vowel syllable and a written symbol consisting of a simple digit-letter combination (e.g. /pa/ may be written as 0í). Afterwards, they are presented with new non-words and have to choose the correct spelling from two possibilities.
4. LLAMA_F tests grammatical inferencing. Participants have 5 min to infer syntax and semantics of 20 sentences from their corresponding pictures. In the testing phase they are presented with two sentences, and they have to choose, which one describes a new picture in a grammatically correct way.

For LLAMA_D, scores range from 0 to 75. For the other subtests, scores range from 0 to 100 (Meara, 2005). The total LLAMA score ranges between 0 and 375.

### Participants

Data used in this study were collected by Kepinska (2017a) and part of it was analysed and reported elsewhere (Kepinska et al. 2017a, b, d, 2018, see below). Three-hundred-and-seven participants were recruited at Leiden University for performing the LLAMA test. Two-hundred-and-thirty-nine of them completed all parts of the test and the biographical information sheet, had Dutch as a first language and were not early bilinguals (i.e., did not acquire a second language before the age of four). Early bilinguals were excluded from our study to avoid confounding effects of non-elective bilingualism on aptitude.

### MRI data acquisition

Of the 239 participants above, eighty-two (59 female, 18–43 years old, *M* = 22.83, *SD* = 4.12) right-handed individuals were invited for MRI scanning, on the basis of either high or average scores on the vocabulary (LLAMA_B) or grammar learning (LLAMA_F) subtest (according to the criteria of the larger study for which the data were collected). Brain imaging data were acquired using a Philips 3T MR-system (Best, The Netherlands) at Leiden University Medical Centre (LUMC), equipped with a SENSE 32-channel head coil. For each participant, an anatomical image including a 3D gradient-echo T1-weighted sequence was acquired (TR = 9.755 ms, TE = 4.59 ms; matrix 256 × 256; voxel size: 1.2 × 1.2 × 1.2 mm; 140 slices). Functional and diffusion-weighted data collected within the framework of this project from *n* = 42 participants were described in previous reports (Kepinska et al. 2017a, b, d, 2018). Structural T1-weighted data from all *N* = 82 participants have not yet been used in any other published report.

### Multilingual experience

Participants completed an online questionnaire in which they listed up to five foreign languages and the age at which they acquired them.

To assess multilingual experience in a continuous way, with more weight given to languages learned earlier in life, we created a single "language experience" score per participant (including their first language, following Kepinska et al. 2023). For this purpose, the age of acquisition (AoA) of each language was log-transformed (to minimise the differences between values for languages learned later in life) and inverted (to express early AoAs as the highest values). To avoid values equal to zero, a constant value of 1 was added before each step. The language experience score was calculated using Shannon's entropy (H) equation (Shannon 1948), where *n* stands for the number of languages participants reported, and *p*_*i*_ for the AoA index (as a proportion of all languages' AoA indices).

$$H=-{\sum }_{i=0}^{n}{p}_{i}{log}_{2}{p}_{i}$$

It was calculated using the R entropy package (v1.3.1; Hausser and Strimmer 2021). More extensive multilingual experience is expressed by higher language experience values (see visual representation of language experience in Figure S1).

Two participants that were invited for MRI scanning had incomplete questionnaires and were excluded from analyses of multilingualism, resulting a final sample size of N = 80 for this analysis.

### Neuroanatomical measures

T1-weighted MRI images were processed using FreeSurfer, version 7.2 (Fischl et al. 2004). The output of FreeSurfer was then further segmented using the Toolbox for the Automated Segmentation of Heschl’s Gyrus (TASH, Dalboni da Rocha et al. 2020). For the current study, the extended version of TASH, called ‘TASH_complete’, was used. It extracts a numerical output for surface area, average thickness and volume for each TTG of each hemisphere (Dalboni da Rocha et al. 2020). The TTGs segmented by TASH were then visually inspected, and those located fully or predominantly in the parietal extension of the PT (Honeycutt et al. 2000) were removed. If half or two-thirds of the TTG was located on the superior temporal gyrus, the remainder was subtracted from volume and surface area. In two participants, the first left hemisphere TTG selected by TASH actually belonged to the planum polare and was therefore removed. This visual inspection of the TASH output was performed independently by three individuals, and discrepancies were discussed and resolved. Visual inspection resulted in the removal of more TTGs in the right hemisphere than in the left hemisphere (Table 1). In the end, participants had one to four TTGs per hemisphere, which were numbered accordingly. Statistical analyses were performed on first versus second versus third gyrus. Too few people had four gyri.

**Table 1:** Original and adjusted number of TTGs before and after visual inspection, per hemisphere

Number of TTGs	Original		After visual inspection	
	Left hemisphere	Right hemisphere	Left hemisphere	Right hemisphere
1	1	3	2	16
2	24	30	43	53
3	42	41	32	12
4	14	8	5	1
5	1			

The Multivariate Concavity Amplitude Index (MCAI) was calculated on the output of TASH (Dalboni da Rocha et al. 2023). The MCAI calculates a concavity score for each TTG separately in each of four orientations: anterior, posterior, medial, and lateral. In this study, only lateral MCAI values were used since most sulci in CSDs occur laterally (Dalboni da Rocha et al. 2023). Therefore, MCAI scores will refer from here on to lateral MCAI only. The lateral multiplication index per hemisphere was calculated by adding the sum of the MCAI scores to the number of TTGs.

Furthermore, asymmetry indices of all TTGs were calculated for the following variables: TTG volume, area, thickness, lateral multiplication index and number per hemisphere. For this, TTG volume, area, MCAI scores and number per hemisphere were summed and TTG thickness was averaged across the identified gyri per hemisphere. The following formula was used to calculate the asymmetry indices:

$$\frac{(Left-Right)}{(Left+Right)}$$

To correct for volume of the cranium, estimated Total Intracranial Volume (eTIV) as given by FreeSurfer was used as a covariate of no-interest. All statistical analyses were performed using the R Statistical Software (v4.1.1; R Core Team 2021).

### Data analysis

Our analysis can be separated into two parts: first we focused on the relationship between language aptitude and TTG anatomy, and second, we explored a relationship between those measures and multilingual experience.

We used mixed models for all our initial analyses to establish (1) which measures (area, thickness, volume), pertaining to (2) which gyri (1st, 2nd 3rd)) were associated with language aptitude or multilingual language experience. The mixed models are used for analyses of data that are hierarchically organized, in our case where measures of different subregions from each participant can be included, and the fact that they are not independent from one another can be accounted for. The linear models were run in follow-up analysis, to gain further insight into the magnitude and direction of the established relationships, whenever the data lacked the hierarchical structure (i.e., measures of one gyrus only were included in the models).

To analyse the relationship between total LLAMA score and TTG structure, we first ran linear mixed-effect models of TTG volume, surface area, and thickness. Based on their results, we ran linear models between specific TTG structural measures, total LLAMA score, and LLAMA subtests. To test our prediction of a more complex multiplication pattern of TTGs with higher language aptitude, we ran a linear model between first MCAI score, lateral multiplication index, and total LLAMA score. To understand if asymmetry between hemispheres influences language aptitude, we ran linear models between asymmetry indices and total LLAMA score.

To investigate the relationship between multilingual experience and auditory cortex anatomy, we first ran a linear model between total LLAMA score and language experience measures and then ran a mixed effect model of TTG volume, surface area, and thickness. Table 2 presents an overview of all performed analyses. Further details are presented in the text.

**Table 2:**
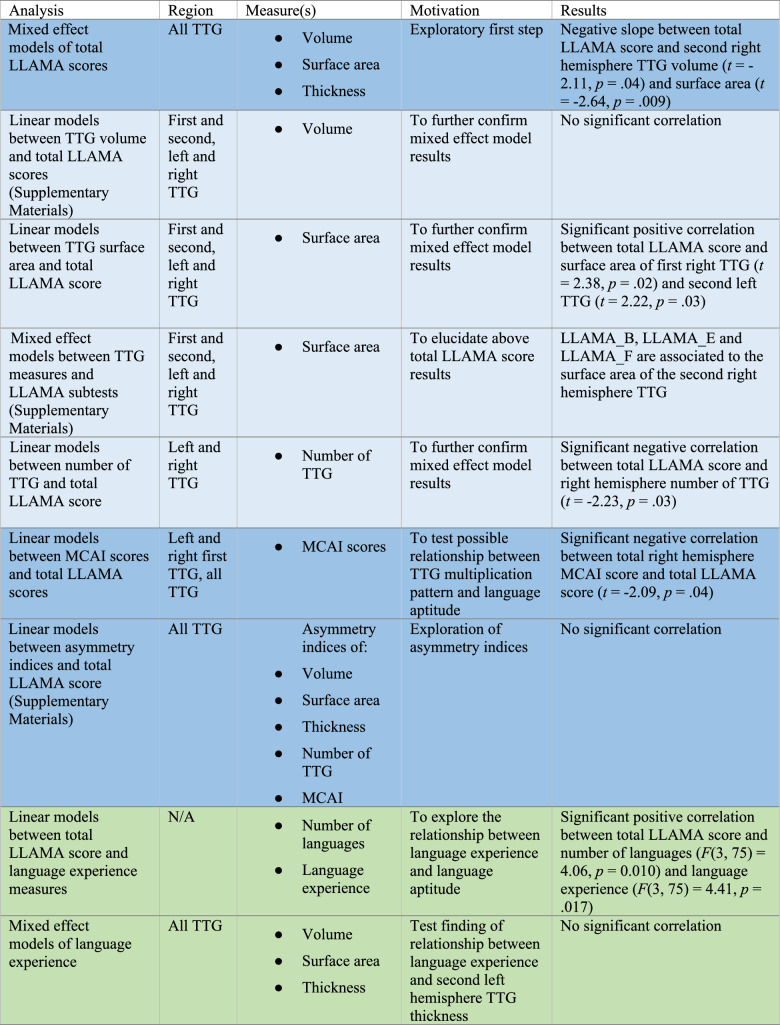
Overview of neuroanatomical and behavioural analyses

Dark blue indicates primary analyses, which were corrected for multiple testing, and lighter blue indicates follow-up analyses. Green indicates analyses in relation with multilingual experience

## Results

### Language aptitude

**Fig. 2:**
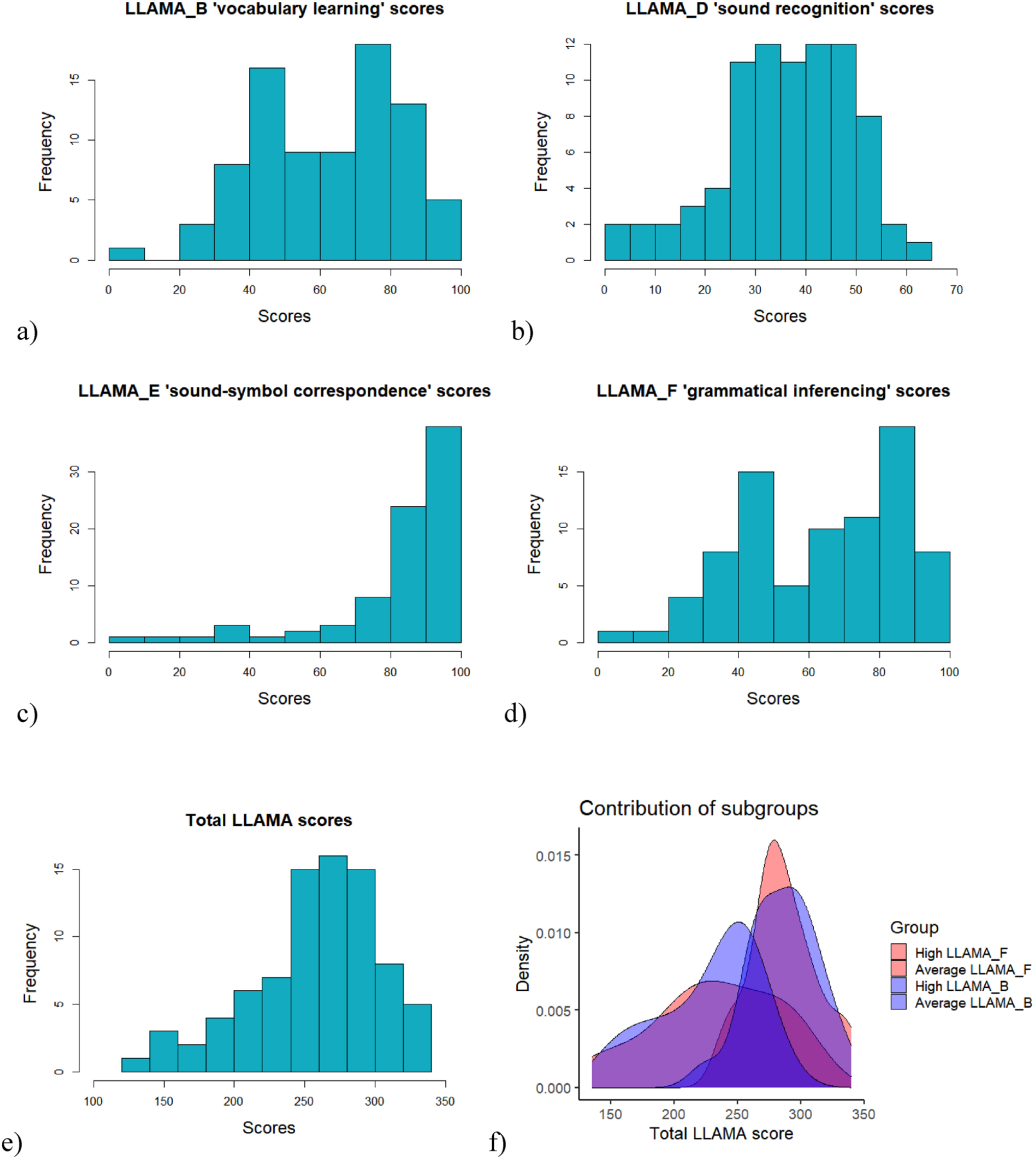
**a–e** LLAMA subtests and total LLAMA score, **f** density plot of subgroup contribution to total LLAMA score

Total LLAMA scores ranged from 135 to 340 (*M* = 258.60, *SD* = 46.00). The total LLAMA score combines the scores of LLAMA_B (*M* = 64.45, *SD* = 20.89), LLAMA_D (*M* = 38.66, *SD* = 13.13), LLAMA_E (*M* = 87.20, *SD* = 20.08), and LLAMA_F (*M* = 68.29, *SD* = 23.08). In general, the scores for the subtests and total LLAMA were high (Fig. 2). This is probably due to the high level of education of the participants; as university students, they are already a sample selected for academic achievement, which includes language skills. This is particularly evident in LLAMA_E: the sound-symbol correspondence task, which shows a ceiling effect. LLAMA_B and LLAMA_F are bimodal because participants were selected for imaging based on average and high scores on these tests. Due to the variability in performance across the subtests, we initially focused on the total LLAMA score, which reflects an average of different language learning subskills, but we also subsequently examined the subtests separately.

### Mixed effect models of language aptitude and TTG structural features

In an exploratory analysis, see Table 2 (1), we performed three linear mixed effects analyses of the relationship between TTG volume, surface area, average thickness and language aptitude using the R package lme4 (Bates et al. 2015). As fixed effects, we considered age, sex, estimated total intracranial volume (i.e. to control for global differences), and the interaction between total LLAMA scores, number of gyrus and hemisphere. We included intercepts for subjects as random effects. Visual inspection of the residual plots revealed no obvious deviations from homoscedasticity or normality. Results are shown in Table S1 in the supplementary materials.

There was a significant interaction between total LLAMA score and volume and surface area of the second TTG, the right hemisphere, and the second right hemisphere TTG.

The Benjamini–Hochberg correction for multiple comparisons (Benjamini and Hochberg 1995) was applied to all effects with total LLAMA score of this model, as well as to models between total LLAMA score and auditory cortex multiplication pattern and asymmetry. All previously significant comparisons with surface area remained significant, while those with volume did not.

### Language aptitude and TTG surface area

To explore the significant interactions with TTG volume and surface area of the linear mixed effect analyses, we ran follow-up linear models. As cortical volume is a measure reflecting both surface area and cortical thickness and volume was not significant after correction for multiple comparisons, we focused on area. For results on volume (analysis (1a) in Table 2), see Table S2 and Figure S2 in the Supplementary Materials. The results of linear models between total LLAMA score and the first and second right and left hemisphere TTG area (analysis (1b) in Table 2) are reported in Table 3.

**Table 3:** Results of linear models with total LLAMA score as the dependent variable and left and right first and second TTG surface area as the explanatory variable

		Total LLAMA score			
		1st TTG		2nd TTG	
		Left	Right	Left	Right
(Intercept)	β	293.262***	276.979***	254.686**	291.897**
	*SE*	(81.435)	(79.755)	(82.756)	(92.426)
Estimated Total Intracranial Volume	β	0.000	0.000	0.000	0.000
	*SE*	(0.000)	(0.000)	(0.000)	(0.000)
Sex	β	0.791	6.196	6.017	2.356
	*SE*	(13.890)	(13.776)	(13.900)	(15.927)
Age	β	– 0.523	– 1.585	– 0.827	– 0.738
	*SE*	(1.338)	(1.298)	(1.288)	(1.636)
**TTG Area**	**β**	**– 0.078**	0.150*	0.127*	**0.074**
	***SE***	**(0.063)**	**(0.063)**	**(0.057)**	**(0.087)**
Num.Obs		82	82	80	66
*R*^*2*^		0.028	0.077	0.069	0.023
*R*^*2*^ Adj		– 0.023	0.029	0.020	– 0.041
AIC		869.2	865.0	845.6	704.6
BIC		883.6	879.4	859.9	717.8
Log.Lik		– 428.597	– 426.485	– 416.798	– 346.319
RMSE		45.05	43.91	44.30	45.99

The bold variable is the variable of interest

**Fig. 3:**
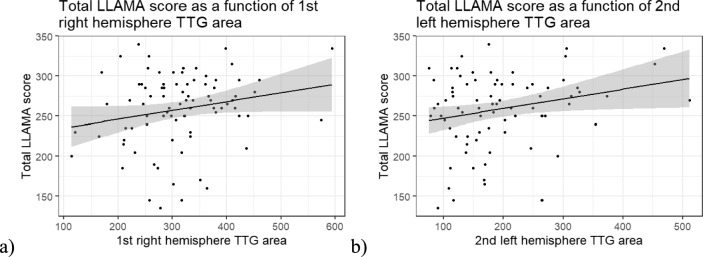
Scatterplots of total LLAMA score and **a** first right hemisphere TTG surface area and **b** second left hemisphere TTG surface area

The overall regression between the first right hemisphere TTG area and total LLAMA score was not significant (Adj. *R*^*2*^ = 0.03, *F*(4, 77) = 1.60, *p* = 0.183) (Fig. 3a). However, the variable of interest alone, first right hemisphere TTG area, had a statistically significant positive relationship with the total LLAMA score (*β* = 0.15, *SE* = 0.06, *t* = 2.38, *p* = 0.020).

The overall regression between the second left hemisphere TTG area and total LLAMA score was not significant (Adj. *R*^*2*^ = 0.02, *F*(4, 75) = 1.40, *p* = 0.242) (Fig. 3b). However, the variable of interest alone, second left hemisphere TTG area, had a statistically significant positive relationship with the total LLAMA score (*β* = 0.13, *SE* = 0.06, *t* = 2.22, *p* = 0.030). The other linear models were not significant (Figure S3 in the Supplementary Materials).

To further explore the relationship between total LLAMA score and TTG surface area, we performed linear mixed effect analyses of TTG surface area for every LLAMA subtest individually (analysis (1c) in Table 2). Table S3 in the Supplementary Materials shows which effects were significant. All subtests as well as the total LLAMA score show significant relationships with TTG area except for LLAMA D, the test for phonetic memory.

In summary, there is a positive relationship between the total LLAMA score and the surface area of the first right and the second left TTG and LLAMA_B (vocabulary learning), LLAMA_E (sound-symbol correspondence) and LLAMA_F (grammatical inferencing) have a significant interaction with TTG surface area, but not LLAMA D (sound recognition).

### Language aptitude and number of TTGs

To test our prediction of more TTGs in the right hemisphere being related to higher language aptitude, we ran linear models between total LLAMA score and the number of TTGs in the left and right hemisphere (1d). This was supported by the mixed models, in which all significant effects were located in the right hemisphere. For our linear models we used age and sex as covariates. The results are shown in Table 4.

**Table 4:** Results of linear models with total LLAMA score as the dependent variable and left and right hemisphere number of TTGs as the explanatory variable

		Total LLAMA score	
		Left hemisphere	Right hemisphere
(Intercept)	β	287.435**	349.035***
	*SE*	(85.764)	(82.138)
Estimated Total Intracranial Volume	β	0.000	0.000
	*SE*	(0.000)	(0.000)
Sex	β	0.976	– 0.276
	*SE*	(14.109)	(13.575)
Age	β	– 0.768	– 1.407
	*SE*	(1.334)	(1.291)
**Number of TTGs**	**β**	**4.680**	– 18.213*
	***SE***	**(8.288)**	**(8.181)**
Num.Obs		82	82
*R*^*2*^		0.013	0.069
*R*^*2*^ Adj		– 0.038	0.020
AIC		870.4	865.7
BIC		884.9	880.1
Log.Lik		– 429.220	– 426.832
RMSE		45.40	44.09

The bold variable is the variable of interest

The linear model for the number of gyri in the left hemisphere was not significant (Adj. *R*^*2*^ = 0.02, *F*(4, 77) = 0.25, *p* = 0.907) (Figure S4 in the Supplementary materials).

**Fig. 4:**
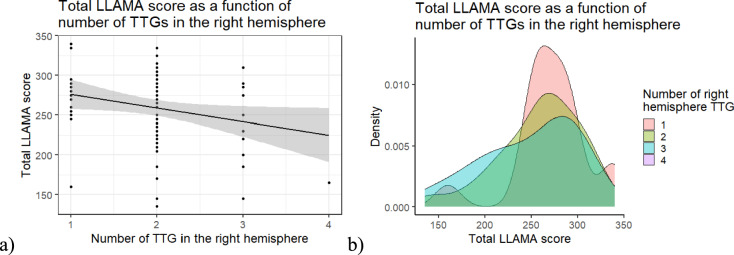
**a** Scatterplot of total LLAMA score and number of TTGs in the right hemisphere **b** Density plot of total LLAMA score and number of TTGs in the right hemisphere

The overall regression of the number of gyri in the right hemisphere was not significant, either (Adj. *R*^*2*^ = 0.02, *F*(4, 77) = 1.42, *p* = 0.235). However, total LLAMA scores had a statistically significant negative relationship with the number of gyri in the right hemisphere (*β* = – 18.21, *SE* = 8.18, *t* = -2.23, *p* = 0.029) (Fig. 4a). Figure 4b shows the distribution of the total LLAMA scores as a function of the number of TTGs in the right hemisphere. In summary, people with one TTG in the right hemisphere are more likely to have a high LLAMA score.

### Language aptitude and TTG multiplication pattern

As TASH does not distinguish between a single HG and a CSD, only the MCAI score of the first TTG can be used for this. For this reason, we ran linear models between the total LLAMA score and the MCAI score of the first TTG and of all TTG, which is the lateral multiplication index (analysis (2) in Table 2) in the left and right hemisphere. Age and sex were included as covariates. Results can be seen in Table 5.

**Table 5:** Results of linear models with total LLAMA score as the dependent variable and left and right first and lateral multiplication index as the explanatory variable

		Total LLAMA score			
		1st TTG		All TTG	
		Left	Right	Left	Right
(Intercept)	β	271.457***	280.269***	255.176***	315.503***
	*SE*	(39.119)	(39.068)	(50.103)	(43.401)
Sex	β	3.187	3.314	4.222	2.788
	*SE*	(12.087)	(11.853)	(12.240)	(11.676)
Age	β	– 0.769	– 1.479	– 0.670	– 1.112
	*SE*	(1.286)	(1.351)	(1.299)	(1.252)
**MCAI score**	**β**	– **11.140**	**62.354**	**4.322**	– 17.224*
	***SE***	**(55.240)**	**(44.264)**	**(8.169)**	**(8.236)**
Num.Obs		82	82	82	82
*R*^*2*^		0.007	0.031	0.010	0.059
*R*^*2*^ Adj		– 0.031	– 0.006	– 0.028	0.023
AIC		868.9	866.9	868.7	864.5
BIC		881.0	878.9	880.7	876.5
Log.Lik		– 429.463	– 428.454	– 429.337	– 427.247
RMSE		45.53	44.98	45.46	44.32

The bold variable is the variable of interest of every linear model

**Fig. 5:**
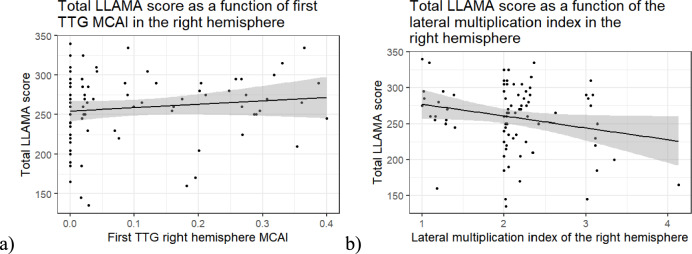
Scatterplot of total LLAMA score and **a** first TTG right hemisphere MCAI **b** total right hemisphere MCAI

The overall regression of the left hemisphere first TTG MCAI (Adj. *R*^*2*^ = – 0.03,* F*(3, 78) = 0.19,* p* = 0.906) (not shown) and right hemisphere first TTG MCAI on the total LLAMA score was not significant (Adj. *R*^*2*^ = – 0.01,* F*(3, 78) = 0.84,* p* = 0.477) (Fig. 5a). The overall regression of the left hemisphere lateral multiplication index (Adj. *R*^*2*^ = – 0.03,* F*(3, 78) = 0.27,* p* = 0.850) and right hemisphere lateral multiplication index (Adj. *R*^*2*^ = 0.02,* F*(3, 78) = 1.64,* p* = 0.187) on the total LLAMA scores was not significant, either. However, the variable of interest, right hemisphere lateral multiplication index, had a statistically significant negative relationship with the total LLAMA score (β = – 17.22, *SE* = 8.24, *t* = -2.09, *p* = 0.040) (Fig. 5b), indicating that higher language aptitude is related to fewer gyri in the right superior temporal plane and to a less complex multiplication pattern of the TTG. This relationship is not significant after correction for multiple testing. It is, however, in accordance with the negative correlation of the number of right hemisphere TTG on total LLAMA score, since the cumulative MCAI score is strongly driven by the number of TTG.

In addition to the first part of our analysis, we explored if language aptitude is related to structural asymmetry of the TTG. We ran linear models between language aptitude and the asymmetry indices of the auditory cortex structural features (analysis (3) in Table 2) that previously showed significant correlations (area, number of TTG, all TTG MCAI) with the total LLAMA score. Age and sex were treated as covariates. Results are reported in Table S4 and Figure S5 of the Supplementary Materials. There were no significant results.

### Multilingual experience

After having analysed the relationship between language aptitude and the anatomy of the auditory cortex, we wanted to relate these variables to multilingual experience. First, we tested our prediction that language aptitude is related to multilingual experience.

**Fig. 6:**
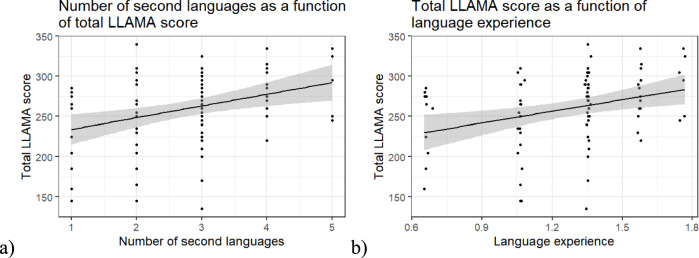
Linear model of **a** number of second languages and **b** language experience as a function of total LLAMA score

The number of second languages that participants reported knowing ranged from one to five, with most participants having learned three (*M* = 2.75, *SD* = 1.07). A linear model with age and sex as covariates was used to determine the effect of the number of second languages learned in life on language aptitude (analysis (4a) in Table 2). The overall regression was statistically significant (Adj. *R*^*2*^ = 0.11, *F*(3, 75) = 4.06, *p* = 0.010) (Fig. 6a). To take also into account age of acquisition for every language, we ran a linear model with age and sex as covariates between our composite language experience index and language aptitude. The overall regression was also statistically significant (Adj. *R*^*2*^ = 0.09, *F*(3, 75) = 4.41, *p* = 0.017) (Fig. 6b). The results are shown in Table 6.

**Table 6:** Results of linear models with total LLAMA score as the dependent variable and number of second languages and language experience as the explanatory variables

		Total LLAMA score	
		Number of second languages	Language experience
(Intercept)	β	212.497***	187.506***
	*SE*	(40.110)	(44.559)
Sex	β	16.546	15.631
	*SE*	(11.717)	(11.819)
Age	β	– 1.380	– 1.287
	*SE*	(1.218)	(1.228)
**Measure**	**β**	17.650***	57.863***
	***SE***	**(4.784)**	**(16.804)**
Num.Obs		80	80
*R*^*2*^		0.160	0.143
*R*^*2*^ Adj		0.126	0.109
AIC		834.4	835.9
BIC		846.3	847.9
Log.Lik		– 412.180	– 412.971
RMSE		41.82	42.23

p < 0.1, * p < 0.05, ** p < 0.01, *** p < 0.001

To determine whether multilingual experience is also related to auditory cortex anatomy, we performed three linear mixed effects analyses on the relationship between TTG volume, surface area, average thickness, and language experience (analysis (4b) in Table 2). As fixed effects, we entered age, sex, estimated total intracranial volume, and the interaction between language experience, number of gyrus, and hemisphere. As random effects, we had intercepts for subjects. Visual inspection of residual plots did not reveal any obvious deviations from homoscedasticity or normality. There was no significant interaction in either mixed model (Table S5 in the Supplementary Materials).

In summary, while there is a significant relationship between total LLAMA and number of second languages and language experience, we found no link to structural measures of the auditory cortex.

#### Does TTG mediates the relationship between language aptitude and multilingual experience?

It is plausible that the significant relationship between the LLAMA scores and multilingual language experience are mediated by the structure of the TTG. To test this hypothesis, we fit three mediation models (using the *lavaan* package in R), with LLAMA total scores as the independent variable, multilingual language experience as the dependent variable and the three TTG characteristics established to be associated with LLAMA scores (1. surface of 1st right gyrus; 2. surface of the 2nd left gyrus; and 3. shape of all right gyri) as mediators, with participants’ age, sex and hemispheric surface area as covariates. All three models resulted in significant total effects (1st right area: ß = 0.331, *p* = 0.001; 2nd left area: ß = 0.330, *p* = 0.001; all right shape: ß = 0.319, *p* = 0.002) and insignificant indirect effects (1st right area: ß = 0.016, *p* = 0.291; 2nd left area: ß = 0.007, *p* = 0.756; all right shape: ß = -0.078, *p* = 0.239) pointing to a lack of mediation of the TTG structure on the language aptitude – multilingual language experience relationship.

## Discussion

In this study, we investigated relationships between the structural features and multiplication patterns of auditory cortex TTGs of participants with differing degrees of language aptitude and with variable multilingual experience. To summarize our results, we found a positive correlation between language aptitude and the surface area of the first right and second left TTG and a negative correlation with number of TTGs in the right hemisphere. All LLAMA subtests were related to TTG surface area except for the subtest of phonetic memory. Neither volume, nor MCAI or asymmetry scores of previously significant measures were significant after correction for multiple testing. The number of languages learned and a summary language experience measure accounting for AoA of each language had a positive correlation with language aptitude, but no relation to structural measures of the TTGs.

### Language aptitude and auditory cortex neuroanatomy

Our main question was the relationship between neuroanatomy and language aptitude, as measured by four subtests of the LLAMA test. We used both the total LLAMA score as the main measure, as it provides an average of specific language learning skills, and individual tests in the follow-up analyses.

We predicted that participants with higher language aptitude would have a more complex multiplication pattern of the TTGs of the right auditory cortex given that Turker et al. (2017) found a positive relationship between language imitation ability and the number of TTGs in the right hemisphere in adults, and Turker et al. (2019) found a positive relationship between language aptitude and the number, CSD and volume of TTGs in the right hemisphere in children and adolescents. However, we found the opposite: participants with high language aptitude had fewer TTG multiplications in the right auditory cortex. This relationship was not found when looking at the shape of HG alone, but only with the multiplication pattern of all TTGs. This relationship was also found when looking at the number of TTGs, a measure that is related to the multiplication pattern of all TTGs, within each hemisphere.

Our results are consistent with previous studies having shown leftwards structural lateralisation of language skills in the auditory cortex: phoneticians are more likely to have multiple or split TTGs in the left auditory cortex (Golestani et al. 2011), as are people who are better at learning to hear foreign speech sounds (Golestani et al. 2007) and lexical tone learners (Wong et al. 2008). However, direct comparison of our study with previous ones may be difficult. Historically, the high variability of TTG's structural features (i.e. volume, surface area, etc.) and multiplication pattern has required manual classification/labelling (Benner et al. 2017; Golestani et al. 2007, 2011; Marie et al. 2015; Schneider et al. 2002, 2005; Turker et al. 2017, 2019). This is prone to error, depends on somewhat inconsistent definitions, and TTG triplications or further multiplications were often not assessed. Here, we used an automated toolbox to continuously assess multiplication pattern variation, which may better detect the presence of more shallow gyri. There may be a difference in the distribution of the number of TTGs in the participant samples of different studies. In our study, few participants had a single gyrus in either the left or right hemisphere. Instead, most participants had at least two TTGs on each side or more, and they had more TTGs in the left hemisphere. In comparison, in the study by Altarelli et al. (2014), which had a similar sample size to ours, all participants had bilateral single gyri except for five who had two TTGs in the right hemisphere. Marie et al. (2015) reported that out of 430 individuals, the most common TTG multiplication pattern combination was bilateral single TTG (36%), with single left and two right TTGs being the second most common (27%) (note that they did not examine additional TTGs). The data from Turker et al. (2017, 2019) also follow this distribution, and Turker et al. (2019) also consider more than two TTGs. Studies using TASH and MCAI show distributions that are more similar to what we found in our study, i.e., more TTGs on the left, and most participants having at least two gyri bilaterally (*n* = 650, unpublished data, Arato 2023).

Our findings that language aptitude is positively correlated with the surface area of the first right and second left TTG and negatively correlated with the number of right TTGs partially align with previous research on individual differences in auditory cortex anatomy and language skill, expertise and disorder. Although it is not easy to reconcile findings in HG vs PT (especially given the inconsistent definitions of the boundary between these, and given the different roles of these likely more primary versus secondary auditory cortex regions in more acoustic versus phonetic processing, respectively), in surface area versus other structural features (e.g. thickness), in shape (i.e. multiplications), and in lateralization, here we venture to propose possible interpretations, which remain to be tested in future studies. First regarding TTGs multiplication patterns, previous work has shown that in dyslexia, there is a higher likelihood of full posterior duplications in children in the right hemisphere (Altarelli et al. 2014; Serrallach et al. 2016), although an older study found this to be the case in the left hemisphere in adults (Leonard et al. 2001). A recent longitudinal study in children, some of whom had a family risk for dyslexia, showed that higher left TTG multiplication patterns predicted better word reading (Blockmans et al. 2023), a finding replicated by Kepinska, Bouhali et al. (2024). On the other hand, work on language has shown a greater likelihood of more TTG in the left hemisphere of people who are good (Golestani et al. 2007) or experts (Golestani et al. 2011) at hearing speech sounds. Here, we found fewer TTGs in the right hemisphere to be related to higher language aptitude. Together, this converges with previous findings in suggesting that higher multiplications in the left TTG and/or fewer ones in the right are indicative of higher language aptitude/skill, and that conversely, more gyri on the right could be a marker for deficit, at least in the domain of language. It is also possible that additional TTG in the right hemisphere would contribute to an overall relatively more right-lateralized PT volume, therefore contributing to a reversal of the known normative leftwards PT volume asymmetry in dyslexia (see below).

Turning to our surface area results, previous studies on language skill, aptitude and expertise used voxel based morphometry or manual labeling to extract HG structural measures, and to our knowledge all only examined volume as a structural measure. Higher volume arises from relatively higher surface area and/or higher thickness. Regarding the exact localization of our results (first TTG in the right and second TTG in the left hemisphere), previous work has shown higher volume of the first TTG in relation to phonetic skill (Golestani et al. 2007; Wong et al. 2008), and a VBM result localized the volume difference in relation to phonetic expertise to the 2nd left TTG (Golestani et al. 2011). Our finding of higher surface area in the first right TTG being related to higher language aptitude is somewhat less expected in terms of lateralization, but nonetheless aligns with findings of higher volume of the first TTG in phonetics experts (Golestani et al. 2011) and in musicians (Schneider et al. 2002).

Direct comparisons between language skill and deficit (i.e. such as dyslexia) should, however, be treated very cautiously because first, many dyslexia studies are done in children whereas the ones on language skill and aptitude are done in adults, and also because post mortem work has shown neural ectopias and cytoarchitectonic abnormalities in dyslexia (Galaburda et al. 1985; Humphreys et al. 1990), which could change the way in which existing HG and additional TTG function alone and in concert with other parts of the language network. Future studies in dyslexic individuals in whom language aptitude has also been tested will be useful for pinpointing similarities and differences in terms of anatomical features underlying speech skill and disorder, as well as the neurofunctional mechanisms underlying the relationships between different anatomical features. For example, although one can speculate that larger surface area (i.e. within one gyrus) could be beneficial to behavior, itis known that sulcal boundaries (i.e. as exist in the case of multiplications) are associated with underlying microstructural and/or cytoarchitectonic differences (Fischl et al. 2008; Welker 1990), and these may lead to beneficial or detrimental effects on auditory and/or higher-level language learning and processing.

In summary, the reason why more gyrification in the auditory cortex leads to worse language aptitude could be the same as why the first right hemisphere surface area and the second left hemisphere surface area is beneficial: a left-lateralization of the (structurally) PT or (functionally) secondary auditory cortex. Increased gyrification could interrupt or limit the auditory processing in the right auditory cortex, which is characterized by lower density of dendrites and axons, but more overlapping dendrites than on the left, is adapted to the integration of relative longer periods of time while on the left axons innervate a smaller number of microcolumns according to a ‘small-world functional network’ structure, which allows fine temporal distinctions (Ocklenburg et al. 2018; Poeppel 2003; Seldon 1981, 1988; Warrier et al. 2009; Zatorre et al. 2002).

We found no evidence for relationships between language aptitude and structural asymmetry indices of all TTGs, either in terms of surface area, number or multiplication pattern, bearing in mind that for all these scores we summed the measures across the gyri belonging to each hemisphere. The reason we found more relationships between language aptitude and right-hemispheric measures may be because the right hemisphere is known to be more anatomically variable (see Dalboni da Rocha et al. (2020), Penhune et al. (1996), Westbury (1999) for reports of greater morphological variability in right HG and STG), and it may be that this variability allows for greater sensitivity in picking up relationships with language aptitude in this sample of average to high language learners.

The relationship between language aptitude and surface area was the same when looking at the total LLAMA score and all the LLAMA subtests except the phonetic memory test (LLAMA_D). It is surprising that of all the tests, the phonetic memory test is not related to auditory cortex surface area. It may be that LLAMA_D, which assesses memory for unfamiliar words, does not rely as much on phonetic processing as on memory.

The relationship between the multiplication pattern of the TTGs and language aptitude may indicate possible innate, genetic influences on this cognitive ability. The unique folding pattern of the human brain, characterised by convex (gyral) and concave (sulcal) regions, serves to increase surface area (Grasby et al. 2020; Rakic 1988). Their development is explained by the radial unit model (Rakic 1988): after migration to the cortex, stacks of neurons, called ontogenetic columns, become basic processing units in the adult cortex. The number of these columns determines the surface area of each cytoarchitectonic region, while their thickness is determined by the number of cell divisions they produce. During evolution, an increase in the number of radial units leads to an increase in surface area and gyrification. This model is supported by twin studies showing that deeper, ontologically earlier sulci are more heritable (Lohmann 1999; Schmitt et al. 2021) and by a modern genome wide association study (GWAS) showing a higher genetic component to cortical surface area, while cortical thickness was found to be more influenced by regulatory elements in the adult brain (Grasby et al. 2020).

An important limitation of our study was the selection bias towards a high level of education of participants, which only allowed comparisons between average and high language aptitude. Thus, our findings are only valid for the high end of language aptitude and results could have been different had people with low aptitude been included. Another limitation is that language aptitude was only measured by the LLAMA test. However, a meta-analysis by Li (2015) showed that language aptitude test scores are indeed positively correlated with final L2 proficiency, and independent of factors such as motivation. Rogers et al. (2017) also found that LLAMA test scores were robust to background variables, with the only limitations being that participants with prior L2 instruction scored higher than monolinguals, and younger children scored lower than adults (Rogers et al. 2017). This strengthens the validity of our results, as our sample included no monolinguals and a relatively narrow age range. The meta-analysis also suggests that the cognitive abilities measured by language aptitude tests are particularly important in early learning stages and in explicit instructional settings, though they lack construct validity (Li 2015).

### Language aptitude and multilingual experience

We discovered a positive association between speakers’ multilingual experience and their language aptitude. Previously, Turker et al. (2017) reported no correlation between the number of languages spoken by adults and their language aptitude. However, Turker et al. (2019) discovered a positive correlation between the number of second languages learned by children and their language aptitude. This finding supports either the conceptualization of language aptitude as an expertise that can be developed based on a predisposition (Grigorenko et al. 2000) or that individuals with a greater aptitude for language seek out more opportunities to learn. While our dataset does not allow us to distinguish between these two possibilities, it is particularly interesting to note that the participants in this study are Dutch, who do not face pressure to learn additional languages beyond English and thus may be closer to elective multilinguals.

We predicted a correlation between auditory cortex thickness and the languages learnt, as Kepinska et al. (2023) discovered a connection between the degree of language experience and the thickness of the second TTG. However, we failed to prove this prediction as there was no significant correlation found between the participants’ language experience and their auditory cortex thickness, or any other measure. This may be due to the exclusion of early bilinguals from our sample, meaning that none of our participants had to accommodate additional languages during the period of life when the brain is highly plastic. Alternatively, our Dutch university participants, who were relatively homogeneous, may not have had sufficient exposure to language diversity compared to the sample of Kepinska et al. (2023). We also did not find a correlation or indirect mediation effect between the auditory cortex surface area and the degree of multilingualism, which would have been indicative of genetic effects on multilingualism mediated by auditory cortex structure. We therefore postulate that the relationship between language aptitude and multilingual language experience could be mediated by structure of a different brain region, beyond the auditory cortex areas investigated in the present study.

### Conclusion

What are the anatomical characteristics of the auditory cortex that relate to language learning talent? Our findings indicate that higher language aptitude correlates with fewer TTGs in the right hemisphere and with greater surface area of the first right and second left TTG. High language aptitude is also associated with having learned multiple languages throughout life.

Future studies on brain anatomy and language aptitude should ideally utilise sizeable datasets for studying and contrasting various populations to discover more subtle relationships between auditory cortex anatomy, behavioral measures, and genetics. Given that language aptitude can be measured relatively easily with the LLAMA test, it could be included as a behavioral measure in large MRI studies to refine our understanding of the relationship between global measures and subcomponents of language aptitude and the anatomy of the auditory cortex. Additionally, by associating knowledge about brain anatomy with genetics, we could evaluate the impact of genetic markers on auditory cortex anatomy and language aptitude. This could provide insight into the degree to which language aptitude is influenced by innate predisposition. Investigating individuals with a wide range of multilingual experience could aid in gauging the effectiveness of language learning in improving language aptitude. It would be worthwhile to investigate whether a relationship exists between language aptitude and the number of languages in populations where learning multiple languages is compulsory, as for instance, in Switzerland or South Tyrol. In addition, the number of foreign languages a person has learned is not necessarily an indication of the person’s level of proficiency. To further explore this question, future studies should evaluate the proficiency level of each spoken language.

Unravelling the relationships between variations in language ability and disability, and the anatomy, function, and genetics of the brain can improve our understanding of how these abilities evolved in our past. Selection works on variation, and by understanding how genetics, brain anatomy, and environmental experiences give rise to variation in language ability, we can try to understand how increasingly higher abilities were selected for in our past.

## Supplementary Information

Below is the link to the electronic supplementary material.

Supplementary file1 (DOCX 253 kb)

## Data Availability

Data will be shared by the corresponding author upon request.
