# Correction: Language aptitude is related to the anatomy of the transverse temporal gyri

**DOI:** 10.1007/s00429-025-02898-5

**Published:** 2025-03-18

**Authors:** Carmen Ramoser, Aileen Fischer, Johanneke Caspers, Niels O. Schiller, Narly Golestani, Olga Kepinska

**Affiliations:** 1 Brain and Language Lab, Vienna Cognitive Science Hub, University of Vienna, Vienna, Austria; 2 Max Planck School of Cognition, Max Planck Institute for Human Cognitive and Brain Sciences, Leipzig, Germany; 3 Faculty of Humanities, Leiden University Centre for Linguistics, Leiden University, Leiden, Netherlands; 4 Department of Linguistics and Translation, City University of Hong Kong, Kowloon Tong, Hong Kong SAR; 5 Brain and Language Lab, Department of Psychology, Faculty of Psychology and Educational Sciences, University of Geneva, Geneva, Switzerland; 6 Department of Behavioral and Cognitive Biology, Faculty of Life Sciences, University of Vienna, Vienna, Austria

**Correction: Brain Structure and Function** 10.1007/s00429-024-02883-4

In this article in the sentence beginning ‘In another study, participants with high language…’ the reference ‘(Kepinska et al. 2017)’ should have read ‘(Kepinska et al. 2017a)’.

In the sentence beginning ‘An EEG study further showed…’ the reference ‘(Kepinska et al. 2017)’ should have read ‘(Kepinska et al. 2017c)’.

In the sentence beginning ‘Data used in this study were…’ the references ‘Kepinska (2017)’ and ‘(Kepinska et al. 2017, 2018, see below)’ should have read ‘Kepinska et al. (2017a)’ and ‘(Kepinska et al. 2017a, b, d, 2018, see below)’.

In the sentence beginning ‘Functional and diffusion weighted data…’ the reference ‘(Kepinska et al. 2017, 2018)’ should have read ‘(Kepinska et al. 2017a, b, d, 2018)’.

In the sentence beginning ‘A recent longitudinal study in children…’ the reference ‘Kepinska, Bouhali et al. (forthcoming)’ should have read ‘Kepinska, Bouhali et al. (2024)’.

In the Table 2 of this article, the section numbers were included inadvertently. The old incorrect and the corrected Table 2 are given below.

**Incorrect version of Table 2**

**Table:**
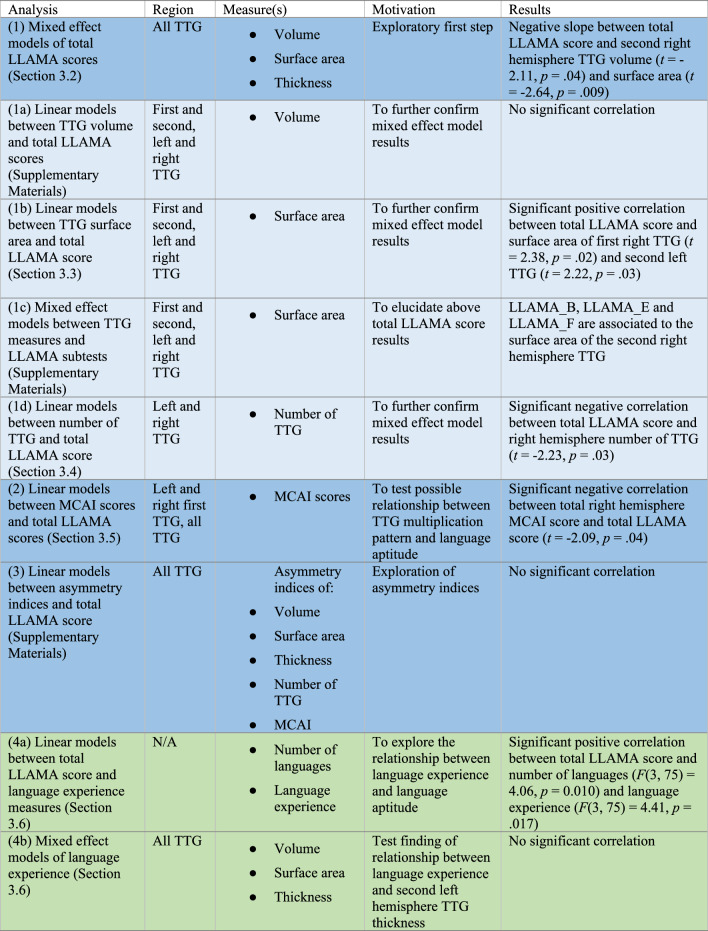
**Table 2** Overview of neuroanatomical and behavioural analyses

**Correct version of Table** 2

**Table 2:**
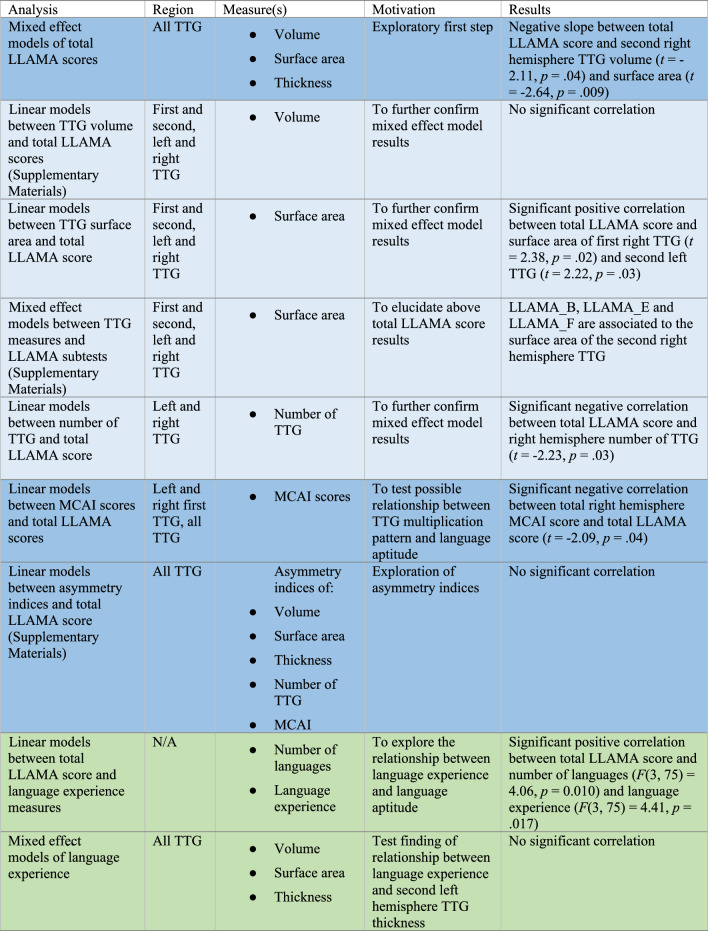
Overview of neuroanatomical and behavioural analyses

Dark blue indicates primary analyses, which were corrected for multiple testing, and lighter blue indicates follow-up analyses. Green indicates analyses in relation with multilingual experience

In this article references pertaining to Kepinska et al. (2017, 2018) were incorrect and should have been as follows.

**Incorrect version of the References:**

Kepinska O (2017) The neurobiology of individual differences in grammar learning. Universiteit Leiden

Kepinska O, Lakke EAJF, Dutton EM, Caspers J, Schiller NO (2017) The perisylvian language network and language analytical abilities. Neurobiol Learn Mem 144:96–101. 10.1016/j.nlm.2017.07.003

Kepinska O, De Rover M, Caspers J, Schiller NO (2018) Connectivity of the hippocampus and Broca’s area during acquisition of a novel grammar. Neuroimage 165:1–10. 10.1016/j.neuroimage.2017.09.058

**Correct version of the References:**

Kepinska O, de Rover M, Caspers J, Schiller NO (2017a) On neural correlates of individual differences in novel grammar learning: an fMRI study. Neuropsychologia 98:156–168. 10.1016/j.neuropsychologia.2016.06.014

Kepinska O, de Rover M, Caspers J, Schiller NO (2017b) Whole-brain functional connectivity during acquisition of novel grammar: distinct functional networks depend on language learning abilities. Behav Brain Res 320:333–346. 10.1016/j.bbr.2016.12.015

Kepinska O, Pereda E, Caspers J, Schiller NO (2017c) Neural oscillatory mechanisms during novel grammar learning underlying language analytical abilities. Brain Lang 175:99–110. 10.1016/j.bandl/2017.10.003

Kepinska O, Lakke EAJF, Dutton EM, Caspers J, Schiller NO (2017d) The perisylvian language network and language analytical abilities. Neurobiol Learn Mem 144:96–101

Kepinska O, de Rover M, Caspers J, Schiller NO (2018) Connectivity of the hippocampus and Broca’s area during acquisition of a novel grammar. NeuroImage 165:1–100

In this article reference Kepinska et al. (2024) was missing and should have been as follows.

Kepinska O, Bouhali F, Degano G, Berthele R, Tanaka H, Hoeft F, Golestani N (2024) Intergenerational transmission of the structure of the auditory cortex and reading skills (p. 2024.09.11.610780). bioRxiv. 10.1101/2024.09.11.610780

The original article has been corrected.

