# Supplementary material for: Language aptitude is related to the anatomy of the transverse temporal gyri
Source: Brain Struct Funct. 2024 Dec 19;230(1):14. doi: 10.1007/s00429-024-02883-4 (PMC11659347; doi:10.1007/s00429-024-02883-4)
Supplement: Supplementary file 1 — Supplementary file1 (DOCX 253 kb) [file 429_2024_2883_MOESM1_ESM.docx]

# Supplementary Materials


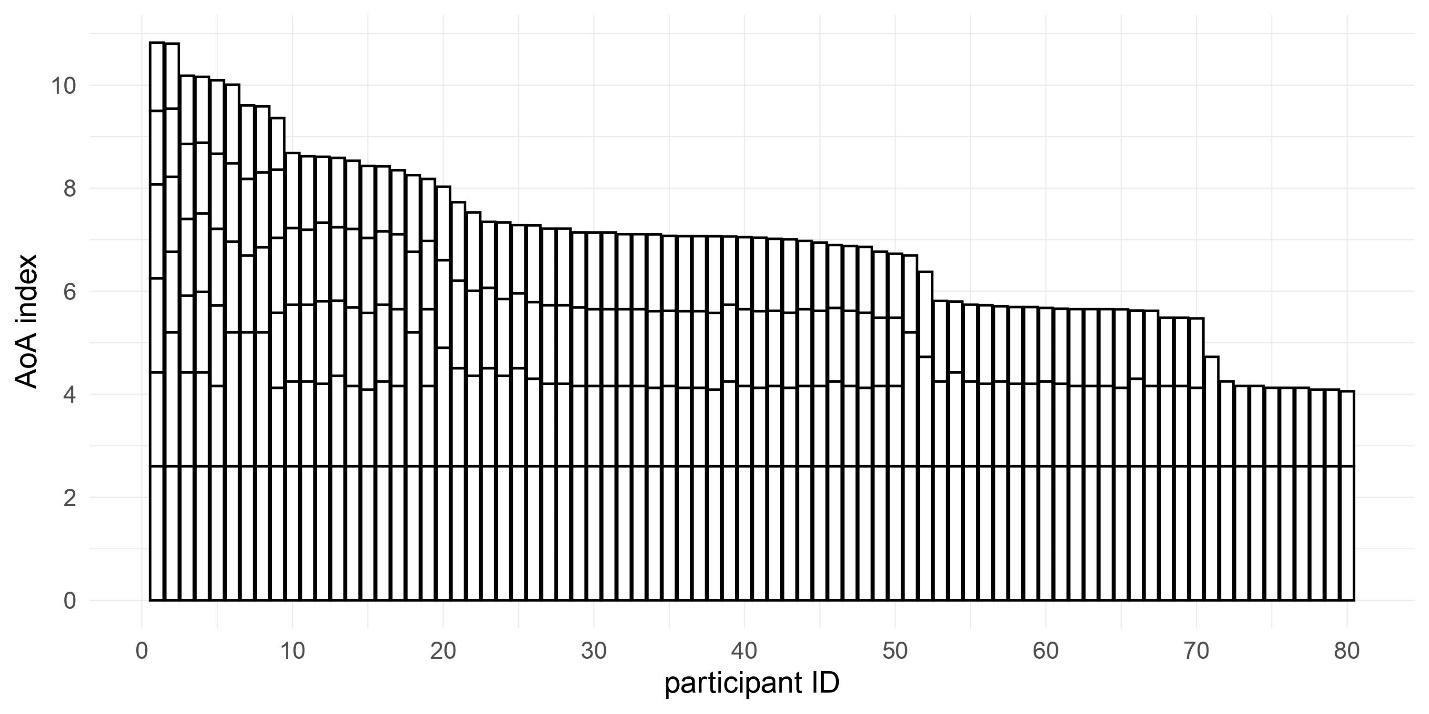


**Fig. S1** Visual representation of language experience sorted by number of languages. Each bar represents a single participant, each stacked bar refers to one language. AoA = Age of Acquisition.

|  |  | Volume | Area | Thickness |
| --- | --- | --- | --- | --- |
| (Intercept) | β | 366.587*** | 0.931*** | -0.086** |
|  | *SE* | (33.978) | (0.078) | (0.026) |
| Age | β | 6.675 | 0.077* | 0.002 |
|  | *SE* | (4.616) | (0.038) | (0.004) |
| Sex 1 | β | -33.346 | 0.006 | -0.074* |
|  | *SE* | (47.737) | (0.103) | (0.033) |
| LLAMA_TOTAL | β | -0.580 | -0.002 | 0.000 |
|  | *SE* | (0.692) | (0.002) | (0.001) |
| Gyrus 2 | β | -516.765*** | -1.363*** | 0.190*** |
|  | *SE* | (42.715) | (0.102) | (0.033) |
| Gyrus 3 | β | -614.602*** | -1.644*** | 0.147*** |
|  | *SE* | (54.801) | (0.130) | (0.042) |
| Hemisphere 1 | β | -154.816*** | -0.343*** | -0.029 |
|  | *SE* | (42.428) | (0.101) | (0.032) |
| LLAMA_TOTAL : Gyrus 2 | β | 2.229* | 0.007** | -0.001 |
|  | *SE* | (0.932) | (0.002) | (0.001) |
| LLAMA_TOTAL : Gyrus 3 | β | 0.705 | 0.002 | 0.001 |
|  | *SE* | (1.227) | (0.003) | (0.001) |
| LLAMA_TOTAL : Hemisphere 1 | β | 1.897* | 0.006** | 0.000 |
|  | *SE* | (0.928) | (0.002) | (0.001) |
| Gyrus 2 : hemisphere1 | β | 38.727 | 0.020 | 0.071 |
|  | *SE* | (62.173) | (0.148) | (0.048) |
| Gyrus 3 : hemisphere1 | β | 28.221 | -0.026 | 0.245** |
|  | *SE* | (103.002) | (0.242) | (0.079) |
| LLAMA_TOTAL : Gyrus 2 : Hemisphere 1 | β | -2.851* | -0.008** | 0.000 |
|  | *SE* | (1.350) | (0.003) | (0.001) |
| LLAMA_TOTAL : Gyrus 3 : Hemisphere 1 | β | -1.959 | -0.007 | 0.000 |
|  | *SE* | (2.027) | (0.005) | (0.002) |
| Cortex Volume | β | 0.002*** |  |  |
|  | *SE* | (0.000) |  |  |
| Cortex White Surface Area | β |  | 0.192*** |  |
|  | *SE* |  | (0.044) |  |
| Cortex Mean Thickness | β |  |  | 1.746*** |
|  | *SE* |  |  | (0.177) |
| SD (Intercept id) |  | 88.735 | 0.139 | 0.083 |
| SD (Observations) |  | 271.591 | 0.647 | 0.208 |
| Num.Obs. |  | 360 | 360 | 360 |
| *R^2^* Marg. |  | 0.489 | 0.569 | 0.426 |
| *R^2^* Cond. |  | 0.538 | 0.588 | 0.505 |
| AIC |  | 5042.4 | 835.4 | 75.3 |
| BIC |  | 5108.5 | 901.4 | 141.4 |
| ICC |  | 0.1 | 0.0 | 0.1 |
| RMSE |  | 256.35 | 0.62 | 0.19 |
| . p < 0.1, * p < 0.05, ** p < 0.01, *** p < 0.001 | | | | |

**Table S1** Results of linear mixed effect models of TTG volume, surface area and average thickness with total LLAMA scores

|  | | Total LLAMA score | | | |
| --- | --- | --- | --- | --- | --- |
|  |  | 1st TTG | | 2nd TTG | |
|  |  | Left | Right | Left | Right |
| (Intercept) | β | 300.524*** | 285.915*** | 273.412** | 289.819** |
|  | *SE* | (81.641) | (80.386) | (81.295) | (92.637) |
| Estimated Total Intracranial Volume | β | 0.000 | 0.000 | 0.000 | 0.000 |
|  | *SE* | (0.000) | (0.000) | (0.000) | (0.000) |
| Sex | β | 0.694 | 3.104 | 4.319 | 2.840 |
|  | *SE* | (13.998) | (13.772) | (13.859) | (15.911) |
| Age | β | -0.787 | -1.202 | -0.919 | -0.718 |
|  | *SE* | (1.318) | (1.290) | (1.281) | (1.640) |
| **Volume** | **β** | **-0.013** | **0.035.** | **0.029.** | **0.017** |
|  | ***SE*** | **(0.017)** | **(0.018)** | **(0.015)** | **(0.023)** |
| Num.Obs. |  | 82 | 82 | 82 | 66 |
| *R^2^* |  | 0.016 | 0.055 | 0.056 | 0.020 |
| *R^2^* Adj. |  | -0.035 | 0.006 | 0.006 | -0.044 |
| AIC |  | 870.2 | 866.8 | 866.8 | 704.8 |
| BIC |  | 884.6 | 881.3 | 881.3 | 718.0 |
| Log.Lik. |  | -429.099 | -427.417 | -427.413 | -346.419 |
| RMSE |  | 45.33 | 44.41 | 44.41 | 46.05 |

**Table S2** Results of linear models of total LLAMA scores depending on left and right first and second TTG volumes. The bold variable is the variable of interest.

a)
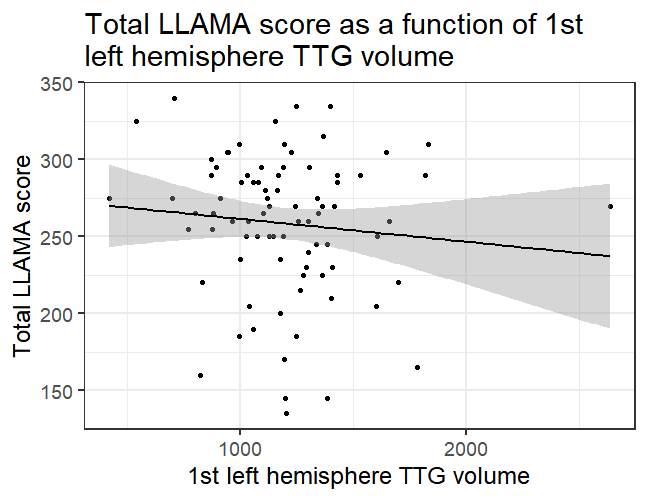
 b)
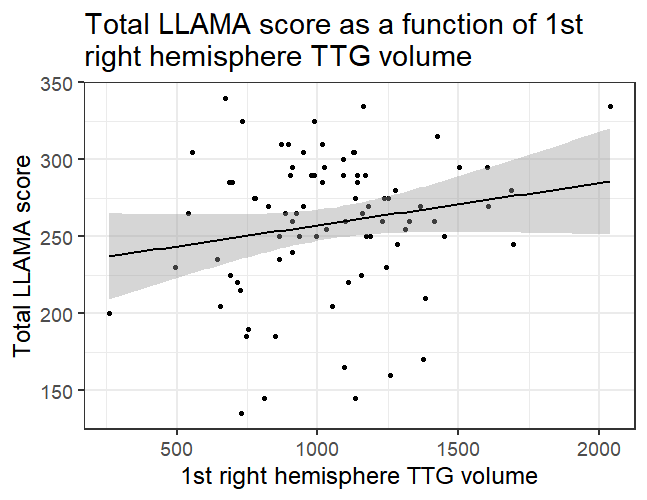


c)
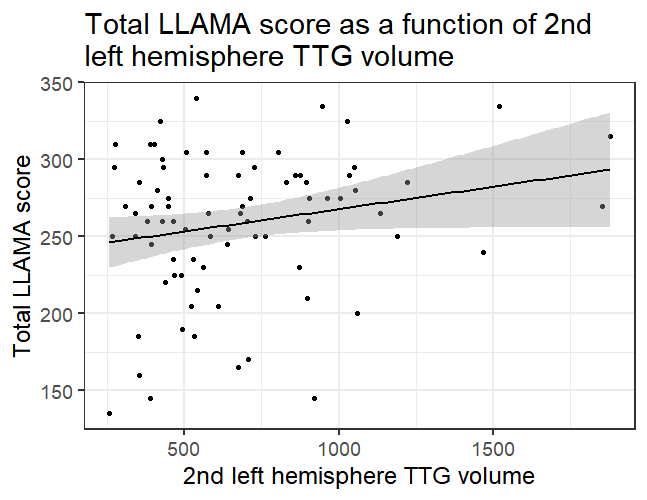
d)
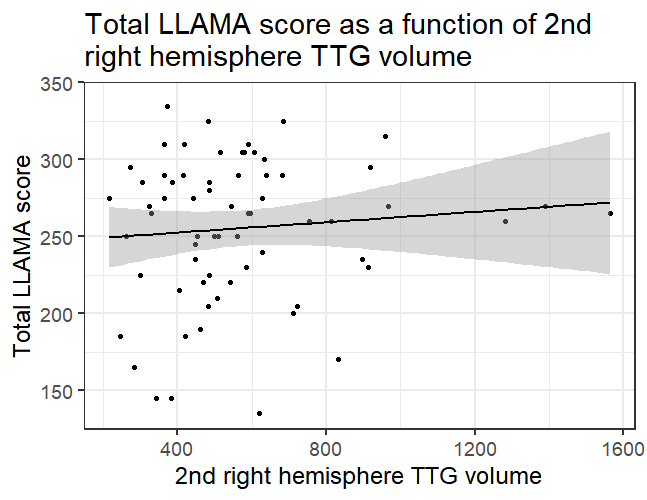


**Fig. S2** **a-d** Scatterplots of total LLAMA score and auditory cortex volumes

a)
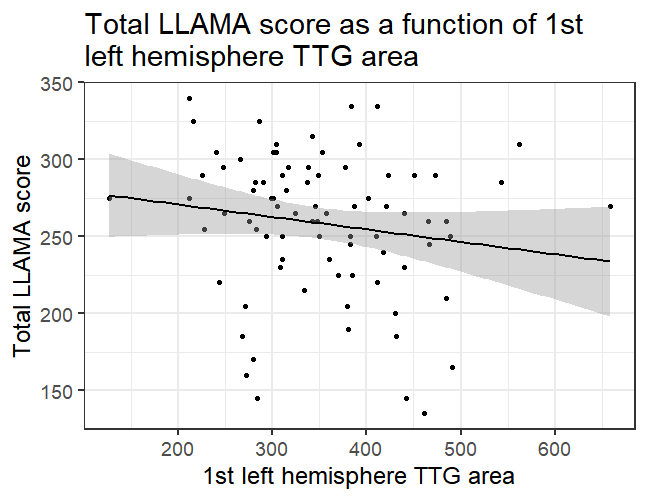
b)
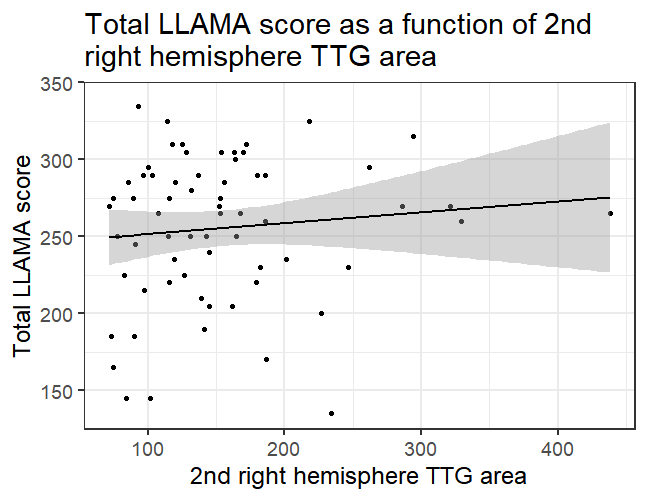


**Fig. S3** Scatterplot of total LLAMA score and **a** first left hemisphere area **b** second right hemisphere TTG area


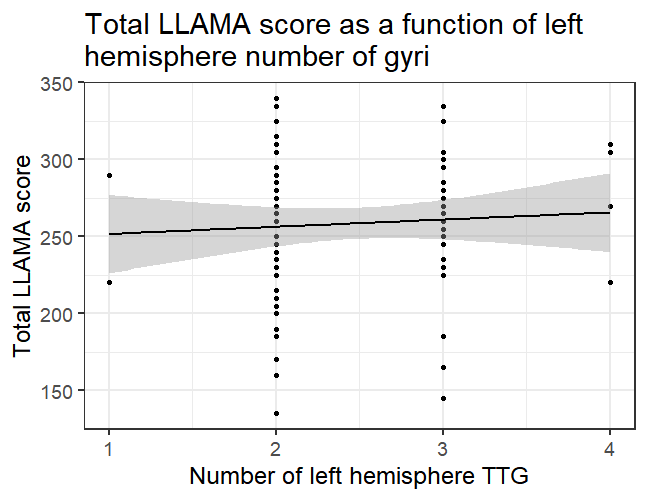


**Fig. S4** Scatterplot of total LLAMA score and number of left hemisphere TTG

|  |  | LLAMA_B | LLAMA_D | LLAMA_E | LLAMA_F |
| --- | --- | --- | --- | --- | --- |
| (Intercept) | β | 0.923*** | 0.927*** | 0.932*** | 0.930*** |
|  | *SE* | (0.079) | (0.080) | (0.079) | (0.078) |
| Age | β | 0.069. | 0.072. | 0.077* | 0.070. |
|  | *SE* | (0.039) | (0.040) | (0.039) | (0.038) |
| Sex | β | 0.034 | 0.009 | -0.001 | 0.003 |
|  | *SE* | (0.106) | (0.107) | (0.104) | (0.103) |
| Surface Area | β | 0.194*** | 0.186*** | 0.194*** | 0.178*** |
|  | *SE* | (0.045) | (0.046) | (0.045) | (0.045) |
| LLAMA_Subtest | β | -0.005 | 0.001 | -0.004 | -0.002 |
|  | *SE* | (0.004) | (0.006) | (0.004) | (0.003) |
| Gyrus 2 | β | -1.361*** | -1.355*** | -1.359*** | -1.358*** |
|  | *SE* | (0.103) | (0.104) | (0.103) | (0.103) |
| Gyrus 3 | β | -1.643*** | -1.641*** | -1.644*** | -1.642*** |
|  | *SE* | (0.131) | (0.133) | (0.132) | (0.131) |
| Hemisphere 1 | β | -0.338** | -0.336** | -0.340*** | -0.340*** |
|  | *SE* | (0.102) | (0.103) | (0.102) | (0.102) |
| LLAMA_Subtest : Gyrus 2 | β | 0.012* | -0.003 | 0.011* | 0.009. |
|  | *SE* | (0.005) | (0.008) | (0.005) | (0.004) |
| LLAMA_Subtest : Gyrus 3 | β | 0.008 | -0.001 | 0.005 | -0.001 |
|  | *SE* | (0.006) | (0.012) | (0.007) | (0.006) |
| LLAMA_Subtest : Hemisphere 1 | β | 0.009. | 0.001 | 0.010* | 0.008. |
|  | *SE* | (0.005) | (0.008) | (0.005) | (0.004) |
| Gyrus 2 : Hemisphere 1 | β | 0.012 | 0.004 | 0.014 | 0.011 |
|  | *SE* | (0.149) | (0.151) | (0.149) | (0.149) |
| Gyrus 3 : Hemisphere 1 | β | -0.013 | -0.003 | -0.004 | -0.031 |
|  | *SE* | (0.238) | (0.241) | (0.245) | (0.252) |
| LLAMA_Subtest: Gyrus 2 : Hemisphere 1 | β | -0.013. | -0.001 | -0.014. | -0.013* |
|  | *SE* | (0.007) | (0.011) | (0.007) | (0.006) |
| LLAMA_Subtest : Gyrus 3 : Hemisphere 1 | β | -0.012 | -0.013 | -0.010 | -0.007 |
|  |  | (0.010) | (0.020) | (0.011) | (0.011) |
| SD (Intercept id) |  | 0.143 | 0.149 | 0.143 | 0.135 |
| SD (Observations) |  | 0.653 | 0.659 | 0.653 | 0.654 |
| Num.Obs. |  | 360 | 360 | 360 | 360 |
| *R^2^* Marg. |  | 0.560 | 0.550 | 0.560 | 0.561 |
| *R^2^* Cond. |  | 0.580 | 0.572 | 0.580 | 0.579 |
| AIC |  | 833.3 | 834.0 | 832.8 | 833.0 |
| BIC |  | 899.4 | 900.0 | 898.9 | 899.0 |
| ICC |  | 0.0 | 0.0 | 0.0 | 0.0 |
| RMSE |  | 0.63 | 0.63 | 0.63 | 0.63 |
| . p < 0.1, * p < 0.05, ** p < 0.01, *** p < 0.001 | | | | | |

**Table S3** Mixed effect model of LLAMA subtest scores depending on surface area measures

|  | | Total LLAMA score | | |
| --- | --- | --- | --- | --- |
|  |  | Area | Number of TTG | All TTG MCAI |
| (Intercept) | β | 266.636*** | 255.836*** | 259.104*** |
|  | *SE* | (40.471) | (38.874) | (38.994) |
| Sex | β | 3.934 | 7.603 | 6.072 |
|  | *SE* | (12.175) | (11.946) | (11.901) |
| Age | β | -0.774 | -0.651 | -0.714 |
|  | *SE* | (1.277) | (1.257) | (1.254) |
| **Asymmetry index** | **β** | **18.165** | **44.883.** | **46.556.** |
|  | ***SE*** | **(37.585)** | **(24.962)** | **(26.292)** |
| Num.Obs. |  | 82 | 80 | 82 |
| *R^2^* |  | 0.010 | 0.046 | 0.045 |
| *R^2^* Adj. |  | -0.029 | 0.009 | 0.008 |
| AIC |  | 868.7 | 844.5 | 865.7 |
| BIC |  | 880.8 | 856.4 | 877.8 |
| Log.Lik. |  | -429.361 | -417.237 | -427.868 |
| RMSE |  | 45.48 | 44.55 | 44.65 |

**Table S4** Results of linear models with total LLAMA score as the dependent variable and asymmetry measures of TTG surface area, number and lateral multiplication index as the explanatory variable. The bold variable is the variable of interest.

The linear model for asymmetry of surface area was not significant before correction (Adj. *R^2^* = -0.03, *F*(3, 78) = 0.25, *p* = .861) (Figure S5a). The linear model for asymmetry of number of gyri was not significant before correction (Adj. *R^2^* = 0.01, *F*(3, 76) = 1.23, *p* = .305). However, there was a tendency towards a relationship with the value of interest (*β* = 44.88, *SE* = 24.96, *t* = 1.80, *p* = .076) (Figure S5b).

The overall regression of lateral multiplication index asymmetry on total LLAMA scores was not significant before correction (Adj. *R^2^* = 0.01, *F*(3, 78) = 1.23, *p* = .307). However, the variable of interest showed a tendency towards a relationship with total LLAMA score (*β* = 46.56, *SE* = 26.29, *t* = 1.77, *p* = .081) (Figure S5c).

a)
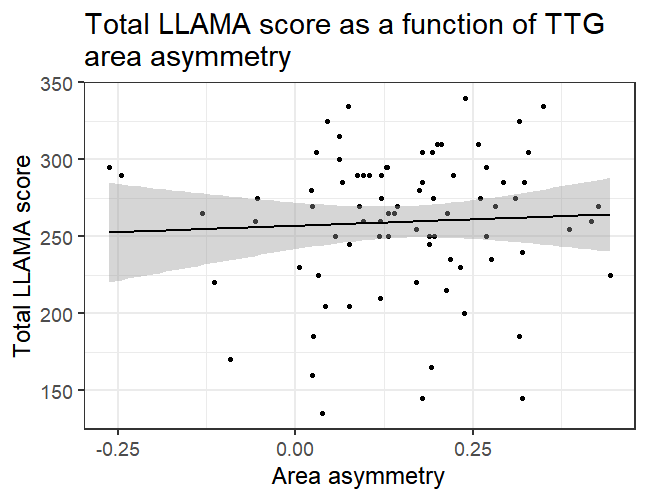
b)
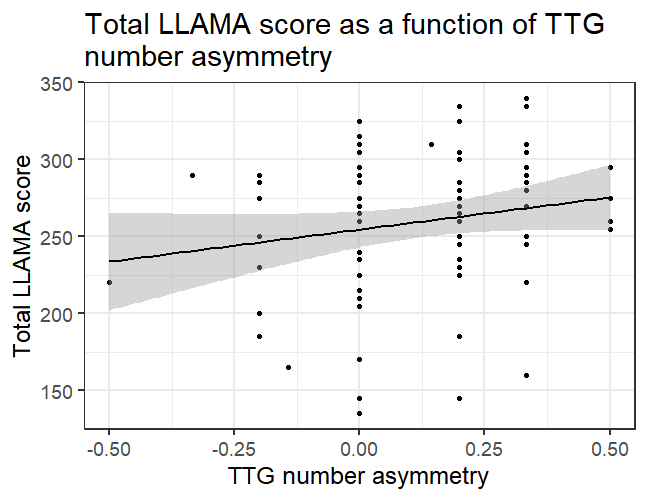


c)
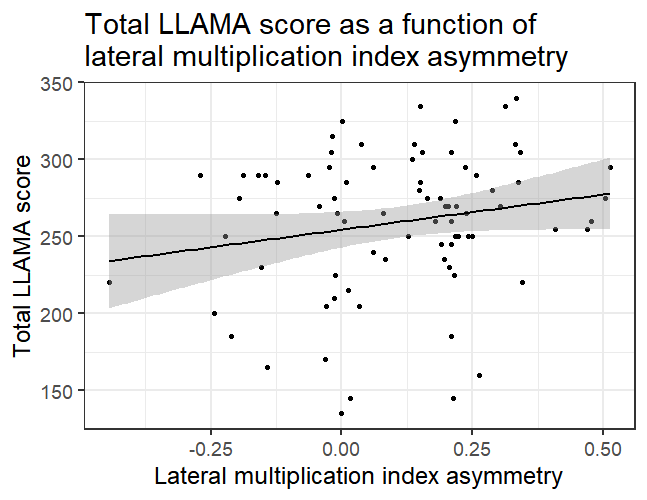


**Fig. S5** Scatterplot of total LLAMA score and **a** TTG area **b** number of TTG asymmetry **c** lateral multiplication index asymmetry

|  |  | Volume | Area | Thickness |
| --- | --- | --- | --- | --- |
| (Intercept) | β | 370.393*** | 0.937*** | -0.083** |
|  | *SE* | (34.082) | (0.079) | (0.026) |
| Age | β | 5.743 | 0.071. | 0.002 |
|  | *SE* | (4.591) | (0.038) | (0.004) |
| Sex 1 | β | -49.549 | -0.026 | -0.085* |
|  | *SE* | (48.313) | (0.105) | (0.034) |
| Language experience | β | 94.219 | 0.061 | 0.009 |
|  | *SE* | (89.879) | (0.073) | (0.069) |
| Gyrus 2 | β | -513.935*** | -1.354*** | 0.189*** |
|  | *SE* | (42.876) | (0.103) | (0.033) |
| Gyrus 3 | β | -612.649*** | -1.640*** | 0.149*** |
|  | *SE* | (54.947) | (0.131) | (0.042) |
| Hemisphere 1 | β | -152.260*** | -0.335** | -0.029 |
|  | *SE* | (42.587) | (0.102) | (0.032) |
| Language Experience : Gyrus 2 | β | -17.944 | 0.003 | -0.019 |
|  | *SE* | (119.013) | (0.100) | (0.090) |
| Language Experience : Gyrus 3 | β | -289.838. | -0.243. | 0.079 |
|  | *SE* | (157.813) | (0.132) | (0.120) |
| Language Experience : Hemisphere 1 | β | 95.252 | 0.078 | 0.071 |
|  | *SE* | (118.563) | (0.100) | (0.090) |
| Gyrus 2 : Hemisphere 1 | β | 29.480 | -0.006 | 0.074 |
|  | *SE* | (62.482) | (0.150) | (0.047) |
| Gyrus 3 : Hemisphere 1 | β | 21.963 | -0.046 | 0.265*** |
|  | *SE* | (101.956) | (0.242) | (0.078) |
| Language Experience : Gyrus 2 : Hemisphere 1 | β | -58.163 | -0.049 | -0.058 |
|  | *SE* | (178.673) | (0.150) | (0.136) |
| Language Experience : Gyrus 3 : Hemisphere 1 | β | 87.433 | -0.013 | 0.449. |
|  | *SE* | (330.191) | (0.274) | (0.252) |
| Cortex Volume | β | 0.002*** |  |  |
|  | *SE* | (0.000) |  |  |
| Cortex White Surface Area | β |  | 0.179*** |  |
|  | *SE* |  | (0.045) |  |
| Cortex Mean Thickness | β |  |  | 1.727*** |
|  | *SE* |  |  | (0.174) |
| SD (Intercept id) |  | 86.935 | 0.134 | 0.079 |
| SD (Observations) |  | 272.668 | 0.654 | 0.207 |
| Num.Obs. |  | 360 | 360 | 360 |
| *R^2^* Marg. |  | 0.487 | 0.560 | 0.435 |
| *R^2^* Cond. |  | 0.534 | 0.578 | 0.507 |
| AIC |  | 4984.7 | 795.4 | 11.4 |
| BIC |  | 5050.8 | 861.5 | 77.5 |
| ICC |  | 0.1 | 0.0 | 0.1 |
| RMSE |  | 257.69 | 0.63 | 0.19 |
| . p < 0.1, * p < 0.05, ** p < 0.01, *** p < 0.001 | | | | |

**Table S5** Results of linear mixed effect models of TTG volume, surface area and average thickness with language experience
